# Functional Evaluation of KEL as an Oncogenic Gene in the Progression of Acute Erythroleukemia

**DOI:** 10.1155/2022/5885342

**Published:** 2022-01-30

**Authors:** Wenjie Liu, Zijuan Wu, Yan Yu, Chun Qiao, Han Zhu, Ming Hong, Yu Zhu, Sixuan Qian, Suning Chen, Depei Wu, Jianyong Li, Hui Jin

**Affiliations:** ^1^Department of Hematology, The First Affiliated Hospital of Nanjing Medical University, Jiangsu Province Hospital, Nanjing 210029, China; ^2^Key Laboratory of Hematology of Nanjing Medical University, Nanjing 210029, China; ^3^Collaborative Innovation Center for Cancer Personalized Medicine, Nanjing 210029, China; ^4^Jiangsu Institute of Hematology, National Clinical Research Center for Hematologic Diseases, the First Affiliated Hospital of Soochow University, Institute of Blood and Marrow Transplantation, Collaborative Innovation Center of Hematology, Soochow University, Suzhou 215000, China

## Abstract

Acute erythroleukemia (AEL) is an infrequent subtype of acute myeloid leukemia (AML) with worse prognosis. Though the last decade has seen major advances in the novel features and genomic landscape in AEL, there is still a lack of specific therapeutic targets and effective treatment approaches for this disease. Here, we found a novel oncogene KEL that specifically and aberrantly expressed in patients with AEL. In this study, we demonstrated that KEL promoted cell proliferation and the downregulation of KEL reversed drug resistance in AEL cells to JQ1. Our findings suggested that KEL contributed to gain of H3K27 acetylation and promoted erythroid differentiation induced by GATA1. Additionally, GATA1 and TAL1 as cotranscription factors (TFs) modulated the expression of KEL. Maintaining cell viability and differentiation, KEL also played parts in the immune evasion of tumor cells. Our work expands the current knowledge regarding molecular mechanisms involved in cancer onset and progression, offering promising therapeutic target to broaden the treatment options.

## 1. Introduction

Leukemia is often originated from certain phases of hematopoietic cells and shows disorders of differentiation. Acute erythroleukemia (AEL) is also named as AML-M6, an invasive form of an infrequent subtype of acute myeloid leukemia (AML) based on the French–American–British (FAB) classification. This disease was first described by Copelli in 1912 [1]. It is characterized by uncontrolled proliferation activity and decreased differentiation capacity of hematopoietic progenitor cells [2]. In recent years, the development and application of deep sequencing technology enable us to have a panoramic view of AML gene mutation spectrum from a macro perspective. Recently, Iacobucci et al. [3] have described the genomic landscape of AEL and identified different recurrent genetic alterations and potential targets for novel therapies. Fagnan et al. [4] also probed the molecular mechanisms and indicated the aberrant activity of key erythroid transcription factors (TFs) in AEL. However, epigenomic heterogeneous architecture and specific targets for the diagnosis and therapy of AEL are in urgent need of exploration.

Erythropoiesis is the process by which hematopoietic cells mature into red blood cells, subject to complex and sophisticated gene expression regulation. Kell blood group (KEL), located on chromosome 7, was initially reported to only expressed in erythroid cells and tissues and appeared prior to erythroid marker GPA [5]. KEL shows extremely high transcriptional activity in erythrocytes whereas basic transcriptional activity in nonerythrocytes [6]. The available data suggests that KEL is highly possible involved in the process of the precise transcriptional regulation and erythroid differentiation.

GATA1, one of the GATA family TFs, has essential roles in hematopoiesis and precise transcriptional regulation process. Expressed in erythroid cells and megakaryocytes, GATA1 is a master regulator of erythroid survival and terminal erythroid differentiation [7]. Not only regulates its own transcriptional activity through posttranslational modification, such as acetylation and phosphorylation, GATA1 can also form dynamic complex with various TFs to selectively regulate downstream genes and participate in erythroid differentiation [8]. Though mounting evidence has indicated GATA1 is one of the prerequisites for leukemogenesis, this factor alone is not sufficient to develop overt leukemias. TAL1 (SCL/TAL1, T-cell acute leukemia protein 1) which encodes a helix-loop-helix transcription factor is also vital in the differentiation of the erythroid lineage [9, 10]. As key erythroid TFs, GATA1 and TAL1, cooperate, along with other proteins, to regulate the process of hematopoiesis and differentiation. And it has been reported that certain erythroid cell-specific genes are activated by a complex formed by GATA1 and TAL1 [11]. The pivotal functional significance of GATA1 and TAL1 in determining lineage fate and lineage-specific differentiation may underlie the pathogenesis of AEL. However, the detail information remains unclear. Recent studies of histone modification have assisted us to better understand the internal regulation of TFs. Controlling gene expression and defining cellular identities, histone modification represents a central oncogenic pathway and drug resistance in AML [12].

Currently, chemotherapy with cytarabine (AraC) and an anthracycline remains the standard treatments in AML including AEL [13]. Molecular biological characteristics are always closely related to the prognosis, and the diagnostic and treatment criteria should be developed accordingly. Insensitivity to chemotherapy, lack of specific therapeutic targets and poor prognosis drive us into in-depth exploration of AEL. It is known that tumor cells have distinct epigenomic characteristics. Multiple evidence has demonstrated BRD4, the epigenetic regulator, as potential therapeutic target in various subsets of AML. JQ1 is a BET bromodomain inhibitor and has been shown to be effective in disrupting the proliferation of tumor cells [14–16]. Although early clinical trials have shown encouraging success of JQ1 treatment in leukemia and lymphoma, drug resistance limits their clinical application [17, 18]. And our knowledge of the molecular mechanisms underlying resistance to JQ1, which is crucial to optimize the clinical efficacy of these drugs, remains incomplete. Therefore, comprehensive studies should be conducted in order to find more specific target. Our findings showed, for the first time, that KEL-mediated epigenetic alterations contribute to JQ1 resistance and provided new insights into the pathogenesis and treatment of AEL.

## 2. Materials and Methods

### 2.1. Patient Samples

Patient samples collected from 31 AML patients and 20 corresponding healthy volunteers between 2017 and 2018 are from the First Hospital Affiliated to Nanjing Medical University and the First Hospital Affiliated to Soochow University. Peripheral blood mononuclear cells (PBMCs) and bone marrow mononuclear cells (BMMCs) were isolated using density gradient centrifugation with Lymphoprep™ (Stemcell technologies, Canada), and written informed consent was obtained from each participant.

### 2.2. Cell Culture and Transfection

Leukemia cell lines K562, NOMO1, and HEL purchased from Cobioer (Nanjing, China) were maintained at 37°C with 5% CO2 in 1640 Medium, supplemented with penicillin/streptomycin (100 *μ*g/ml) (PS, Gibco, Grand Island, USA) and 10% fetal bovine serum (Yeasen, Shanghai, China). HEK-293T was purchased from X-Y Biotechnology (Shanghai, China) and maintained at 37°C with 5% CO2 in DMEM Medium, supplemented with PS, 10% FBS. The cell lines used in this study were authenticated by STR profiling. Lentiviral shRNA vectors were cotransfected into 293T cells with the packaging vectors psPAX2 (Addgene) and pCI-VSVG (Addgene) using a calcium phosphate method to produce viable lentivirus. And KEL overexpressing lentivirus were synthesized by Hanbio Company (Shanghai, China). Cells were transfected with shRNA or the KEL overexpressing lentivirus according to the manufacturer's instructions. Transfection efficiency was determined by real-time quantitative PCR and western blot.

### 2.3. Cell Proliferation Assay

To detect cell proliferation ability, CCK8 (cell counting kit-8) assays were performed, and cells with different treatment were plated in 96 well plates. 10 *μ*l CCK8 solution (Beyotime, Shanghai, China) was added at pointed times; then, the spectrophotometrically at 450 nm was measured by automatic microplate reader (Synergy H1MF; BioTek, Winooski, USA) after incubated at 37°C for 3 h. Experiments were run in triplicate.

### 2.4. Chromatin Immunoprecipitation Assays (ChIP)

ChIP assays were performed using the Magna ChIP A-Chromatin Immunoprecipitation Kit according to the manufacturer's instruction (Cat. 17610, Millipore, USA). The antibodies for Histone H3 (Cat. ab1791) and H3 trimethyl Lys4 (H3K4me3, Cat. ab8580) were from Abcam, and acetyl-histone H3 Lys27 (H3K27Ac, Cat. 4353T) was from Cell Signaling Technology. The ChIP primers were listed in Table. S1. Quantification of immunoprecipitated DNA was performed using qPCR. ChIP data was calculated as a percentage relative to the input DNA from the equation 2[Input Ct − Target Ct] × 100 (%).

### 2.5. Electrophoresis Mobility Shift Assay (EMSA)

To perform the electrophoresis mobility shift assay (EMSA), nuclear extracts were prepared with K562 cells using the NE-PER Nuclear and Cytoplasmic Extraction Reagents (Thermo Scientific). Probes were designed according to previous study [6] and generated by Zoonbio Biotechnology. The sequence of biotin labeled probes are as follows: 5′ GCCACAGAAGATAGACAGATGGTA 3′ (F), 5′ TACCATCTGTCTATCTTCGTGTGGC 3′ (R). Electrophoresis was performed with 8 percent nondenatured polyacrylamide gel, and the gel was dried and subjected to autoradiography.

### 2.6. RNA Isolation and Real-Time Fluorescence RT-qPCR

Total RNA was isolated using the Trizol method (Ambion, USA). cDNA was generated by HiScript reverse transcriptase (Vazyme, Nanjing, China). Gene expression was examined with the Hieff ™ qPCR SYBR Green Master Mix (YEASEN, Shanghai, China). Primers used in this study are listed Table. S1.

### 2.7. Animal Studies

To evaluate the changes of related carcinogenic characteristics of KEL in vivo, 6-week-old male NOD-Prkdc^em26Cd52^Il2rg^em26Cd22^/Nju (T001475) mice were used. Mice were irradiated from X-ray source with a dose of 150 cGy and taken to detect the efficacy of myeloablative treatment. And the bone marrow cells of mice receiving irradiation significantly decreased. The K562 cells (2 × 10^6^/200 *μ*l) with or without KEL overexpression were injected into each irradiated recipient mice through the tail vein in 48 h. PBS was injected as control. The weight and WBCs (white blood cells) were monitored closely. Luciferase signal intensity was detected at week 5. The mice were put to death after they met the euthanasia criteria or bear tumor for 8 weeks; then, the immunohistochemical analysis and H&E staining were performed. Mice were euthanized by intraperitoneal injection of pentobarbital (150 mg/kg i.p.). All animal experiments were approved by the Institutional Animal Care and Use Committee (IACUC, approval No. NRCMM19).

### 2.8. Western Blot

Proteins were obtained from cells lysed in RIPA protein lysate (Beyotime, Shanghai, China) supplemented with protease and phosphatase inhibitors (Roche). Quantification of protein was conducted with Bicinchoninic Acid Protein Assay Kit (Thermo Scientific, USA). Protein samples were separated by 4–12% sodium dodecyl sulfate–polyacrylamide gel electrophoresis (SDS-PAGE) and transferred to polyvinylidene fluoride (PVDF) membranes. Blocked in 5% skim milk for 2 h at room temperature, the membrane was in diluted primary antibody overnight at 4°C. Washing with TBST, the membrane was then incubated with a secondary antibody. The band signals were visualized and quantified using the Quantity One system (Bio-Rad, Hercules, CA, USA). GAPDH and *β*-actin were selected to be the loading controls. All the antibodies employed in this study were listed in Table. S2.

### 2.9. Luciferase Assay

The KEL promoter sequences of 4 segments were synthesized (3 segments 600 bp and 1 segment 500 bp), and the vectors pGL3-KEL1, pGL3-KEL2, pGL3-KEL3, and pGL3-KEL4 were constructed, respectively. The promotor sequences were shown in Table. S3. HEK 293T cell with 70-80% confluency was transfected using Lipofectamine 2000 reagent (Invitrogen, Carlsbad, CA, USA) with the pKEL constructs or an empty pGL3vector, and pcDNA3.1/TAL1 or GATA1 or an empty pcDNA3.1 vector and pRL-TK Renilla luciferase reporter vector (Promega, Madison, WI, USA). Luciferase and Renilla signals were determined 48 h after transfection using the Dual Luciferase Reporter Assay Kit (Promega, Madison, WI, USA) according to a protocol provided by the manufacturer. The relative luciferase activities were calculated based on Firefly/Renilla fluorescence. Three independent experiments were performed, and the data are presented as mean ± SD.

### 2.10. Statistical Analysis

GraphPad Prism 8.0 (GraphPad Software, Inc., La Jolla, CA, USA) was used for data analysis and imaging. Results were presented as the mean ± SD for at least three repeated independent experiments and calculated using Student's *t*-test. In all cases, a *p* value < 0.05 was considered as statistically significant.

## 3. Results

### 3.1. Expression Profile of KEL in AML Patients and Cell Lines

**Figure 1:**
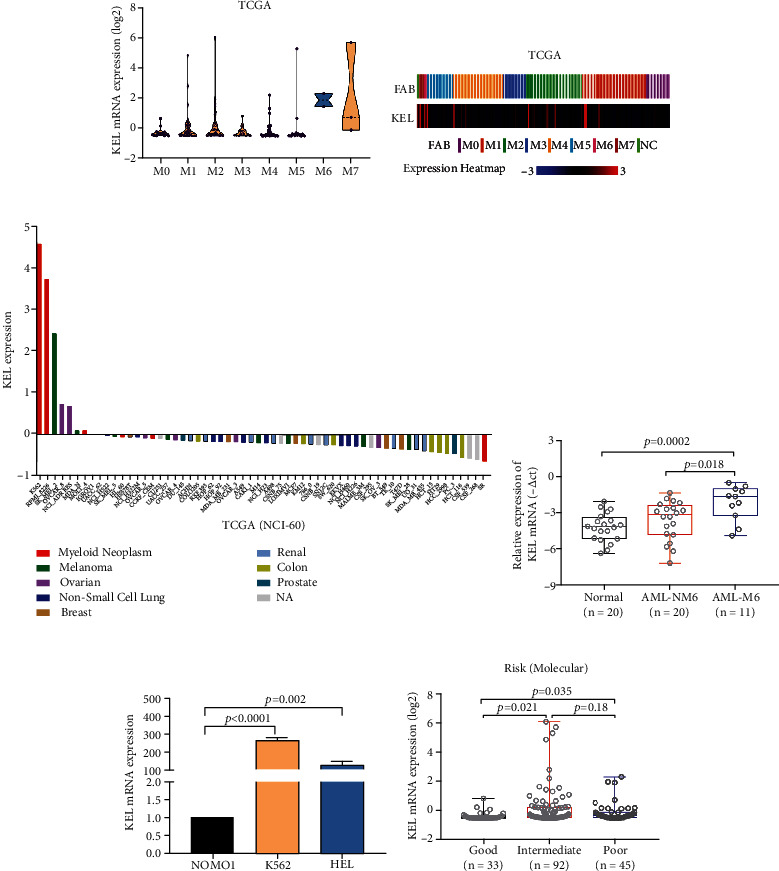
The expression of KEL in AML patients and cell lines. (a, b) The violin figure and heat map exhibiting the expression of KEL mRNA in different subtypes of AML from TCGA database. (c) Expression of KEL in NCI-60 cell lines in TCGA. (d) KEL level detected by qPCR in three leukemia cell lines. (e) The expression of KEL mRNA in healthy volunteers (*n* = 20) and patients with M6 (*n* = 11) or other AML (*n* = 20). (f) The expression of KEL mRNA in all AML patients from TCGA with different molecular risk stratifications.

To better distinguish AEL (M6) from other AML subtypes, we screened The Cancer Genome Atlas (TCGA) database to select specific oncogene in AEL. Using this database, we found that KEL was specifically highly expressed in M6 patients compared with other subtype of AML patients (Figures 1(a) and 1(b)). In addition, M6 cell lines, HEL and K562, showed higher expression of KEL than myeloid neoplasm cell lines (Figures 1(c) and 1(d), Fig. S1A). The potential clinical significance of KEL was then investigated. RT-qPCR and agarose gel electrophoresis analysis demonstrated that, consistent with TCGA database, patients with AML-M6 showed higher level of KEL mRNA compared with non-M6 patients and healthy donors (Figure 1(e) and Fig. S1B). And the level of KEL protein was significantly elevated in AEL patients (Fig. S1B). Further clinical analysis of TCGA database showed that KEL expression level has none business of age and gender (Fig. S1C and D). However, patients presented with intermediate- or poor-risk assessment of molecular have higher KEL level compared to good ones (Figure 1(f)). Combined the clinical characteristics of 20 AEL patients, analysis indicated that there are no significant differences in the levels of white blood cells (WBCs), platelets, and hemoglobin between patients with different KEL expression (Fig. S1E-G). In sum, KEL as a specific indicator in AEL exhibited its potential role in tumorigenesis and serving as diagnosis and prognosis biomarker.

### 3.2. KEL Regulates AEL Cell Proliferation and Its Downregulation Reverses Drug Resistance of JQ1

**Figure 2:**
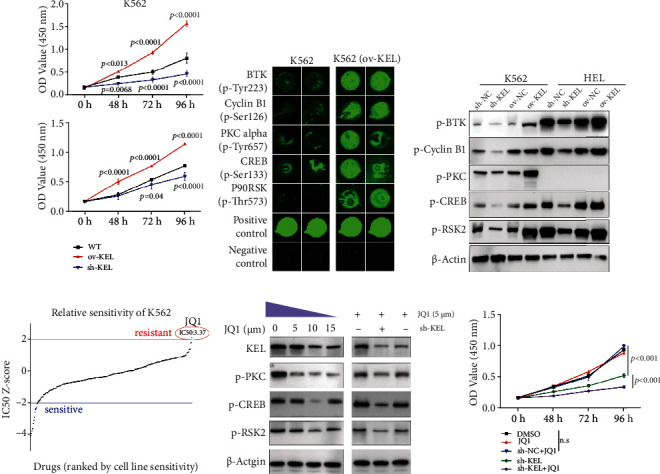
KEL promotes cell proliferation and is responsible to drug resistance to JQ1. (a) Assessment of the proliferation of K562 cells and HEL cells transfected with KEL shRNA or lentivirus-mediated overexpression by CCK-8 assay. Data represents the mean ± SD (*n* = 3). (b) Representative display of dysregulated proteins detected by protein array. (c) Western blot validating the result of protein array. (d) IC50 value showing the drug sensitivity of K562 cells to 318 inhibitors. One resistant inhibitor above the red line and 14 sensitive ones below the blue line (https://www.cancerrxgene.org/). (e) Protein level change induced by JQ1 treatment and/or with KEL downregulation detected by western blot. (f) Knocking down KEL reversed the resistance of JQ1 in K562 as indicated by CCK-8 assays. Data represents the mean ± SD (*n* = 3).

To explore the function of KEL in AEL, we designed three KEL siRNAs and separately transfected K562 and HEL cells and then assessed the knockdown efficiency. Both qRT-PCR and western blot analysis confirmed that siRNA#2 was the most effective for knockdown of KEL expression (Fig. S2A). We then constructed KEL shRNA using the sequence of siRNA#2 to stably knockdown the expression of KEL. And to manipulate the expression of KEL, we succeeded to construct the lentivirus-mediated overexpression (ov-KEL) vectors (Fig. S2B). CCK-8 assay revealed that the K562 and HEL cells in which KEL expression was forced were significantly more likely to exhibit a malignant phenotype than the mock cells. Conversely, reduced KEL expression inhibited the proliferation ability (Figure 2(a)). Next, we performed the protein array to find out abnormally activated pathways induced by KEL. The results were shown in Fig. S2C and Table. S4. And the Fig. S2D showed the overall phosphorylation change level. One key branch signaling pathway (RafB-MEK1-RSK2-CREB) involved in cell proliferation was finally picked out (Fig. S2E). We hypothesized that KEL would serve essential functions in AEL, which depend on the pathway. The expression level of BTK and CyclinB1 was used to confirm the array results (Figure 2(b)). Most importantly, associated genes of the key brunch proposed to be involved in KEL-mediated cell proliferation that was changed correspondingly with KEL up/downregulation, indicating that KEL plays critical roles in AEL cell proliferation (Figure 2(c)).

Currently, the treatment of AEL still follows the common AML therapeutic strategy. Small-molecule inhibitor JQ1 identified to led to robust antileukemic effects has been studied intensively in multiple subtypes of AML [17]. It has been reported that JQ1 is a hopeful choice that targets AML. However, drug resistance occurs frequently, and the mechanism underlying the difference in leukemia stem cell (LSC) sensitivity to JQ1 remains elusive [19]. And forecasting analysis observed that JQ1 treatment seemed to be noneffective in K562 though it exerts an inhibitory effect in multiple cell lines and LSCs [20, 21]. According to the database of all the 318 small-molecule inhibitors, K562 was sensitive to 14 inhibitors and resistant only to JQ1 (Figure 2(d)). Then, we wondered the correlation among KEL, JQ1, and cell proliferation. To figure out whether KEL was associated with the resistance of K562 cells to JQ1, we firstly treated K562 at different dose of JQ1. High dose of JQ1 induced modest decrement of KEL and cell proliferation signal transduction pathway. However, knocking down KEL significantly strengthened the role of JQ1 (Figure 2(e)). CCK8 assay showed that knockdown of KEL forced the role of JQ1, as demonstrated by inhibition of cell proliferation (Figure 2(f)). In K562 cells, inhibition of KEL reversed the relative resistance of JQ1 treatment alone, indicating that KEL plays major roles not only in cell proliferation but also in drug resistance.

### 3.3. KEL Contributes to Gain of H3K27 Acetylation and Promotes GATA1-Induced Erythroid Differentiation

**Figure 3:**
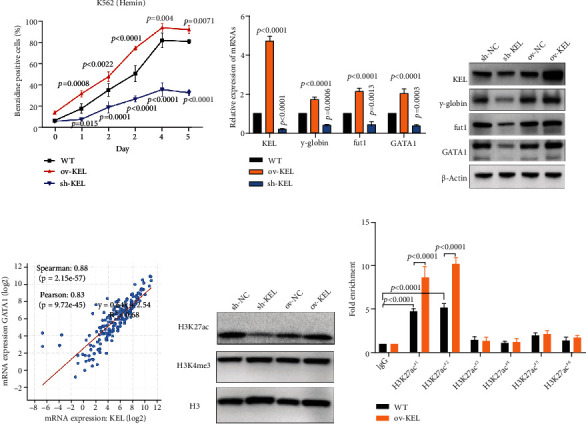
KEL promotes erythroid differentiation and regulates the expression of GATA1 through moderation of H3K27ac. (a) Erythroid differentiation ability induced by Hemin with different KEL level assessed by benzidine staining. (b, c) qPCR and western blot analyzing the expression change of erythroid differentiation marker (*γ*-globin and fut1) and TF (GATA1). (d) Positive relationship between GATA1 and KEL form TCGA. (e) The level of histone with different KEL expressions. (f) The ChIP-qPCR results suggesting most potential modification sites.

Hematopoietic process is the development and mature process of various types of blood cells in human. Erythroid differentiation as an important part plays portal role in AEL. K562 cell line established from a patient with chronic myeloid leukemia (CML) in blast crisis is a classical model to study erythroid differentiation in vitro [22]. Erythroid differentiation of K562 was induced with hemin or sodium butyrate (NaBu) treatment. As it is known that GATA1 is a core TF in erythroid differentiation process [23], we then explored the regulatory effect between KEL and GATA1. Inspiringly, we found that the change of KEL expression could affect erythroid differentiation potential. Upregulation of KEL promoted the erythroid differentiation of K562 cells induced by hemin or NaBu (Figure 3(a) and Fig. S3A). The change of KEL expression led to the corresponding increase or decrease of erythroid differentiation markers (*γ*-globin and fut1) and TF (GATA1) (Figures 3(b) and 3(c)). TCGA database results of the strong positive relevance between KEL and GATA1 indicated the internal regulatory mechanism (Figure 3(d)). Using bioinformatics prediction (http://dbtoolkit.cistrome.org/), we found high regulatory potential of H3K27ac and H3K4me3 at the region of GATA1 near transcription start site in K562 (Fig. S3B). Western blot analysis showed that the level of H3K27ac not H3K4me3 changed with KEL (Figure 3(e)). ChIP-seq exhibited the peak of H3K27ac at GATA1 locus region, and different primers were designed according to various peaks (Fig. S3C). Through ChIP-qPCR analysis, we observed the enrichment of H3K27ac at the promoter region of GATA1 and gain of H3K27ac in KEL overexpressed cells (Figure 3(f)). Taken together, these data confirmed that KEL promoted the gain of histone sites H3K27ac of GATA1 promoter and partially accounted for the significant activation of GATA1 induced erythroid differentiation.

### 3.4. GATA1 and TAL1 as Co-TFs Regulate the Expression of KEL

**Figure 4:**
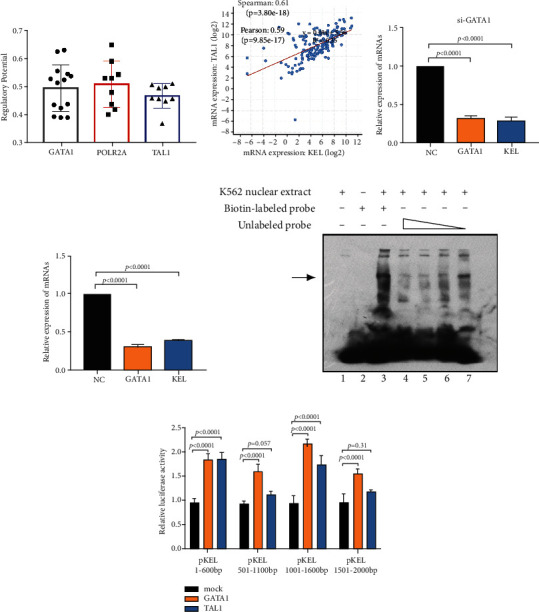
GATA1 and TAL1 are TFs of KEL. (a) The most potential TFs predicted by Toolkit. (b) TCGA analysis of the expression levels between KEL and TAL1. The change of KEL after knocking down (c) GATA1 and (d) TAL1. (e) EMSA result validating the specific binding of KEL and TFs. Each panel represents the results using the corresponding paired of probes as indicated. Lane 1 and lane 2: negative control without nuclear extracts or biotin-labeled probes; lane 3: using nuclear extract with the biotin-labeled probes; lanes 3-7 using unlabeled probes with decreased concentration, respectively, 150x, 100x, 50x, and 10x. (f) The ChIP-qPCR results suggesting most potential modification sites. Data represents the mean ± SD (*n* = 3).

TF networks exert essential roles in erythroid differentiation [24]. Through online database (Cistrome Data Browser), we have selected multiple TFs with high regulatory potential of KEL in K562. GATA1 and TAL1 as famous TFs and POLR2A which encodes the largest subunit of RNA polymerase II showed the highest regulatory potential (Figure 4(a)). We have already showcased the highly positive correlation between KEL and GATA1 (Figure 3(d)).

TCGA database also suggested that patients with higher level of TAL1 seem to possess higher KEL expression (Figure 4(b)). To verify the hypothesis that TAL1 and GATA1 are TFs of KEL, we separately knocked down TAL1 and GATA1 with siRNAs. Results showed that downregulation of GATA1 and TAL1 reduced the expression of KEL (Figures 4(c) and 4(d)). In addition, consistent with previous research [6], EMSA result revealed that K562 nuclear extract could specifically bind to biotin-labeled probe, and the competition occurred after the addition of the cold probe. With the increase of the cold probe concentration, the competition increased (Figure 4(e)). Subsequently, in order to narrow down the area on which GATA1 and TAL1 exerted effects within KEL promoter, dual luciferase reporter assays were performed with truncated segments of KEL. Four shorter fragments with three 600 bp and one 500 bp of the promoter were cloned. The results implied that GATA1 affected all the promoter regions while TAL1 mainly affected the proximal 200 bp (Figure 4(f)). Thus, we concluded that the two TFs, GATA1 and TAL1, directly interact with KEL to coactivate KEL in K562.

### 3.5. KEL Enhances Tumor Cell Proliferation and Tumor Growth In Vivo

**Figure 5:**
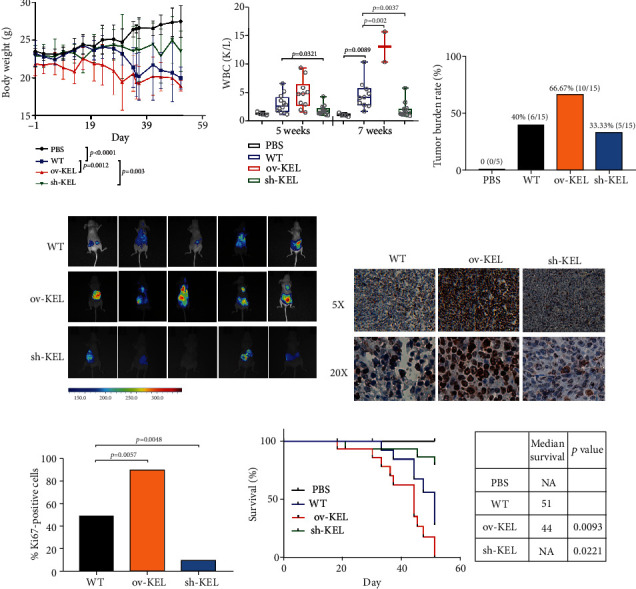
KEL promotes tumorigenesis in vivo. (a) Body weight of mice transplanted with PBS or K562 cells with different treatment. (b) WBC detection at weeks 5 and 7. (c) The tumor burden rate of all the mouse models. (d) Representative images of bioluminescence imaging of AEL mice after vail vein injection at week 6. (e) Immunohistochemistry analysis of ki67 in tumors from AEL mice. (f) ki67 positive ratio calculated and shown in histogram. (g) Kaplan-Meier curves of overall survival showing the difference between mice with low and high KEL levels.

To verify the in vitro findings, we examined the biological functions of KEL in mediating proliferation in vivo. K562 cells with stably forced (ov-KEL group) and decreased KEL (sh-KEL group) expression were transplanted into NCG mice by tail intravenous injection. Phosphate buffered saline was injected for control group (PBS group). Consistent with the above in vitro findings, the overexpression of KEL dramatically promoted AEL progression. 23 days after injection of K562 cells, mice began to lose weight. Weight loss of mice in the ov-KEL group started from 19 days was more dramatic than the WT group and sh-KEL group (Figure 5(a)). By week 5, the differences of white blood cells (WBCs) have turned up in experimental groups. 7 weeks later, 10 mice survived in the WT group and 13 in the sh-KEL group, whereas only 2 survived in the ov-KEL group with extremely high WBC counts (Figure 5(b)). During the growth phase, lumps were observed in the abdominal cavity and hind limbs in AEL mice. Tumor burden rates were calculated, and the tumor formation capability of the ov-KEL group was greater than the WT and sh-KEL group (Figure 5(c)). The growth state of tumors was observed by whole-body fluorescent imaging system at week 6 postinjection (Figure 5(d)). Using bioluminescent imaging, we found that tumors harvested from the ov-KEL group had significantly higher fluorescence signals, and the sh-KEL group had lower fluorescence signals compared with those from the WT group. Immunohistochemical (IHC) assay was used to analyze the tumor biopsy specimen and evaluate the pathological feature. And the results proved that KEL could promote tumor cell proliferation (Figure 5(e)). The histogram visually displayed the ratios and the significant difference of ki67 positive cells between the three groups (Figure 5(f)). Importantly, our results showed that mice transplanted using cells with higher expression of KEL had a significantly worse prognosis. In contrast, knocking down of KEL prolonged the survival of AEL mice, which further verified the role of KEL in AEL (Figure 5(g)). In general, these results demonstrated that KEL enhanced proliferation of tumor cells and was strongly associated with the progression and prognosis of AEL.

### 3.6. PD-L1 Positively Correlates with KEL May Induce Immune Evasion of Tumor Cells

**Figure 6:**
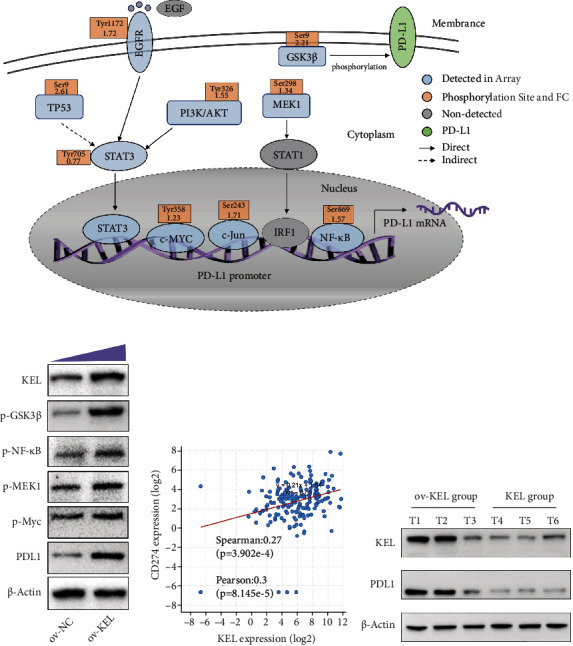
KEL affects the expression of PDL1 and associated genes. (a) Comprehensive showcase of related genes both reported before and detected in our array that moderate the expression of PDL1 (CD274). Fold change was listed in the orange box. (b) Western blot verifying the result in K562 cells. (c) TCGA analysis of the expression of KEL and PDL1. (d) Representative expression of KEL and PDL1 protein level in tumors (T: tumor).

PD-L1 is known to be typically expressed on the surface of tumor cells and allow them to evade the immune system surveillance [25]. Unexpectedly, our data showed that the majority of proteins that reported to be involved in the biological process of PD-L1 were found to be upregulated in K562 cells with KEL overexpression (Figure 6(a) and Table. S4). The results were further verified by western blot which was consistent with protein array. Most importantly, PD-L1, lowly expressed in K562 cells, was significantly upregulated after the enhancement of exogenous expression of KEL (Figure 6(b)). To probe the functional consequences of PD-L1 expression in AEL and the relationship between PD-L1 and KEL, we screened the database and discovered a positive correlation between the two factors (Figure 6(c)). Western blot conducted with total cells isolated from 3 pairs of AEL mice tumors exhibited higher levels of KEL and PD-L1 protein in the ov-KEL group compared with the WT group (Figure 6(d)). Collectively, these results manifested that low expression of PD-L1 in K562 could be enhanced by the upregulation of KEL and perhaps provided possible checkpoint inhibitor therapy strategy for AEL patients.

## 4. Discussion

Over the past decade, we have seen major advances in human gene sequencing for the identification of novel specific oncogenes in multiple tumors. AEL is still a hematologic malignancy that is hard to conquer. For chemotherapy resistance and no targeted drugs, AEL carries a high mortality [26]. Despite recent attempts for novel approaches of molecular inhibitors such as JQ1, drug resistance of leukemic cells still occurs, and the mechanisms that render the phenomenon remain largely undefined [19]. It is in urgent need to elucidate the drug resistance mechanisms and thus excavate novel therapeutic targets. An initial objective of this study was to identify specific carcinogens that contribute to AEL progression and that serve as novel diagnostic and therapeutic molecular markers. Encouragingly, KEL is discovered aberrantly expressed in AEL compared with other subtypes of AML. Being identified to be involved in regulating proliferation and differentiation of AEL cells, high expression of KEL has been proved to accelerate leukemia progression in vivo as well and always indicate poor prognosis.

Genome-wide epigenetic analysis has illustrated that the conformation of chromatin region might be responsible for gene transcription and regulation. The loss or gain of H3K27me and H3K27ac results in gene transcriptional activation or repression [27]. HDACi can regulate the balance between H3K27me and H3K27ac. And several research and clinical trials have reported that HDACis display greater efficacy in multiple tumors [28–30]. But how chromatin structure is regulated in tissue- and differentiation stage-specific patterns and how TFs play roles in the adaptation of chromatin structure are just begin to be explored. Prior studies have noted the importance of GATA1 in the regulation of hematopoiesis and differentiation. However, the complete mechanisms through which GATA1 exerts its functions and how it contributes to epigenetic plasticity in AEL are not well understood. Notably, our subsequent exploration and analysis suggest that KEL is positively correlated to GATA1. The internal relationship between them is then investigated. We report here the oncogenic transcriptional reprogramming mediated by KEL's interaction with GATA1, which directs the erythroid differentiation of AEL cells. Our data shows that GATA1 is enriched for H3K27ac. Consistent with previous report [6], we verified GATA1 as a TF regulating the status of KEL. Moreover, TAL1 is also identified as a co-TF. While whether they act alone or in concert still needs to be explored. Our findings of the positive feedback loop of KEL, H3K27ac, and GATA1 may further enrich and deepen the mechanism by which GATA1 regulates erythroid differentiation. Hence, it could conceivably be hypothesized that reciprocal cross exists, which magnifies the carcinogenesis.

Development of epigenetic modulators holds promise for novel therapeutic interventions [31]. JQ1 involved in transcriptional repression is recently noted to be effective in multiple tumors, including AML [32, 33]. Despite its inspiring efficacy, the drug resistance existed in K562 cells is not ignorable. In the present study, we find a high degree of correlation between KEL and JQ1, supporting our findings that increased KEL negates the effects of JQ1, while the downregulation of KEL significantly reverses the phenomenon. Our research may broaden horizon of the mechanisms underlying the resistance of K562 to JQ1. HDACis also induce chromatin changes which are effective in multiple tumors, especially in hematological tumors [34, 35]. HDACi-Dacinostat suppressed the expression of KEL and GATA1. This phenomenon indicated that HDACi treatment could be potential therapeutic strategy. Notably, KEL is also known as CD238. As a membrane protein, KEL is a promising target for the development of novel therapeutic strategy. In addition, immunotherapy such as PD1, PD-L1 monoclonal antibodies that target immune checkpoints have granted the exceptional edge in leukemias [36–38]. Nevertheless, a great deal of patients poorly responded with no definite reasons. Our observation of KEL-induced high PD-L1 level raises the possibility that KEL might partly explain the response of AEL patients to immunotherapy. However, how KEL modulates PD-L1 expression and their interactions in tumor immune escape still need to be investigated. Since KEL is a membrane protein on the surface of tumor cells, whether its ligands exist in immune cells and what the ligands are? In addition, whether the bridging effect between them can promote disease and affect immunotherapy? There are still many questions worth to be further explored in our future work.

Our research though preliminarily identifies KEL as a clinically relevant marker for diagnosis, prognostication, and disease monitoring in AEL. The finding of a significantly increased expression KEL may provide a rationale therapeutic strategy for AEL patients.

## 5. Conclusions

In general, this study identifies KEL as a novel molecular marker that helps to define the diagnosis and prognosis. Additionally, high expression of KEL induces the resistance of K562 to JQ1. Mechanistically, KEL interacts with two vital TFs: TAL1 and GATA1, promoting cell proliferation and erythroid differentiation. Our work uncovers a critical function of KEL and provides insight into the ideal therapeutic strategy for AEL patients.

## Data Availability

The protein array data used to support the findings of this study are included within the supplementary information file(s).

## Supplementary Materials

Supplementary 1 Additional file: Figure S1: expression of KEL in cell lines and AML patients. (A) KEL expression in different cell lines from Expression Atlas (https://www.ebi.ac.uk/gxa/home). (B) Protein and mRNA level of KEL in healthy volunteers and M6 patients and other AML patients detected by western blot AGE. TCGA analysis of KEL expression of AML patients with different (C) age and (D) gender. The relationship among the expression of KEL and (E) WBCs, (F) Hb, and (G) platelets.

Supplementary 2 Additional file: Figure S2: the results of protein array. (A) The knockdown and (B) overexpression efficacy of KEL in K562 and HEL cells. (C) The exhibition of phosphorylated antibody array. (D) Overall phosphorylated level compared between K562 cells with or without KEL expression. (E) Key brunch signaling pathway picked out that involved in cell proliferation.

Supplementary 3 Additional file: Figure S3: biological and regulatory function of KEL. (A) Benzidine staining of K562 induced with NaBu. (B) Regulatory potential of the GATA1 histone moderation predicted by online database Toolkit for Cistrome Data Browser (http://dbtoolkit.cistrome.org/). (C) ChIP-seq analysis of GATA1 from Toolkit. Design diagram of primers to perform ChIP-qPCR was showed. Data represents the mean ± SD (*n* = 3).

Supplementary 4 Additional file: Table S1: primers used in this study.

Supplementary 5 Additional file: Table S2: antibodies and chemicals used in this study.

Supplementary 6 Additional file: Table S3: promoter sequence of GATA1.

Supplementary 7 Additional file: Table S4: the results of phosphorylated protein array.
