# Supplementary figures and images for: Functional Evaluation of KEL as an Oncogenic Gene in the Progression of Acute Erythroleukemia
Source: Oxid Med Cell Longev. 2022 Jan 30;2022:5885342. doi: 10.1155/2022/5885342 (PMC8819426; doi:10.1155/2022/5885342)

**A**

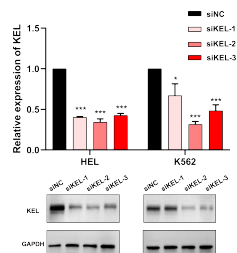

**B**

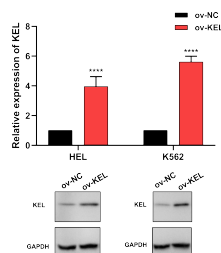

**C**

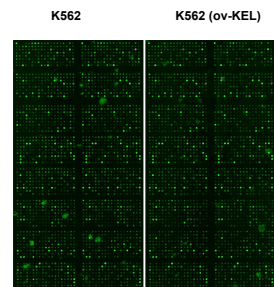

**D**

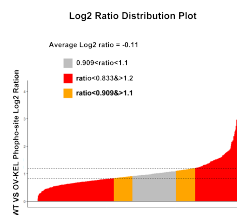

**E**

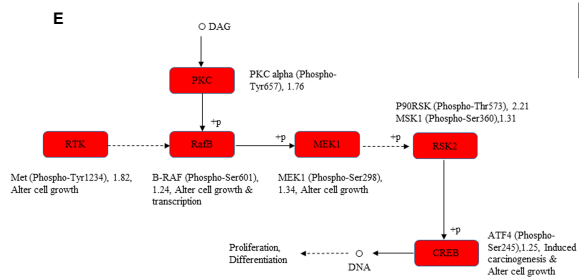

Supplement: Supplementary 2 — Additional file: Figure S2: the results of protein array. (A) The knockdown and (B) overexpression efficacy of KEL in K562 and HEL cells. (C) The exhibition of phosphorylated antibody array. (D) Overall phosphorylated level compared between K562 cells with or without KEL expression. (E) Key brunch signaling pathway picked out that involved in cell proliferation. [file 5885342.f2.pdf]
