# Supplementary material for: Functional Evaluation of KEL as an Oncogenic Gene in the Progression of Acute Erythroleukemia
Source: Oxid Med Cell Longev. 2022 Jan 30;2022:5885342. doi: 10.1155/2022/5885342 (PMC8819426; doi:10.1155/2022/5885342)
Supplement: Supplementary 1 — Additional file: Figure S1: expression of KEL in cell lines and AML patients. (A) KEL expression in different cell lines from Expression Atlas (https://www.ebi.ac.uk/gxa/home). (B) Protein and mRNA level of KEL in healthy volunteers and M6 patients and other AML patients detected by western blot AGE. TCGA analysis of KEL expression of AML patients with different (C) age and (D) gender. The relationship among the expression of KEL and (E) WBCs, (F) Hb, and (G) platelets. [file 5885342.f1.pdf]

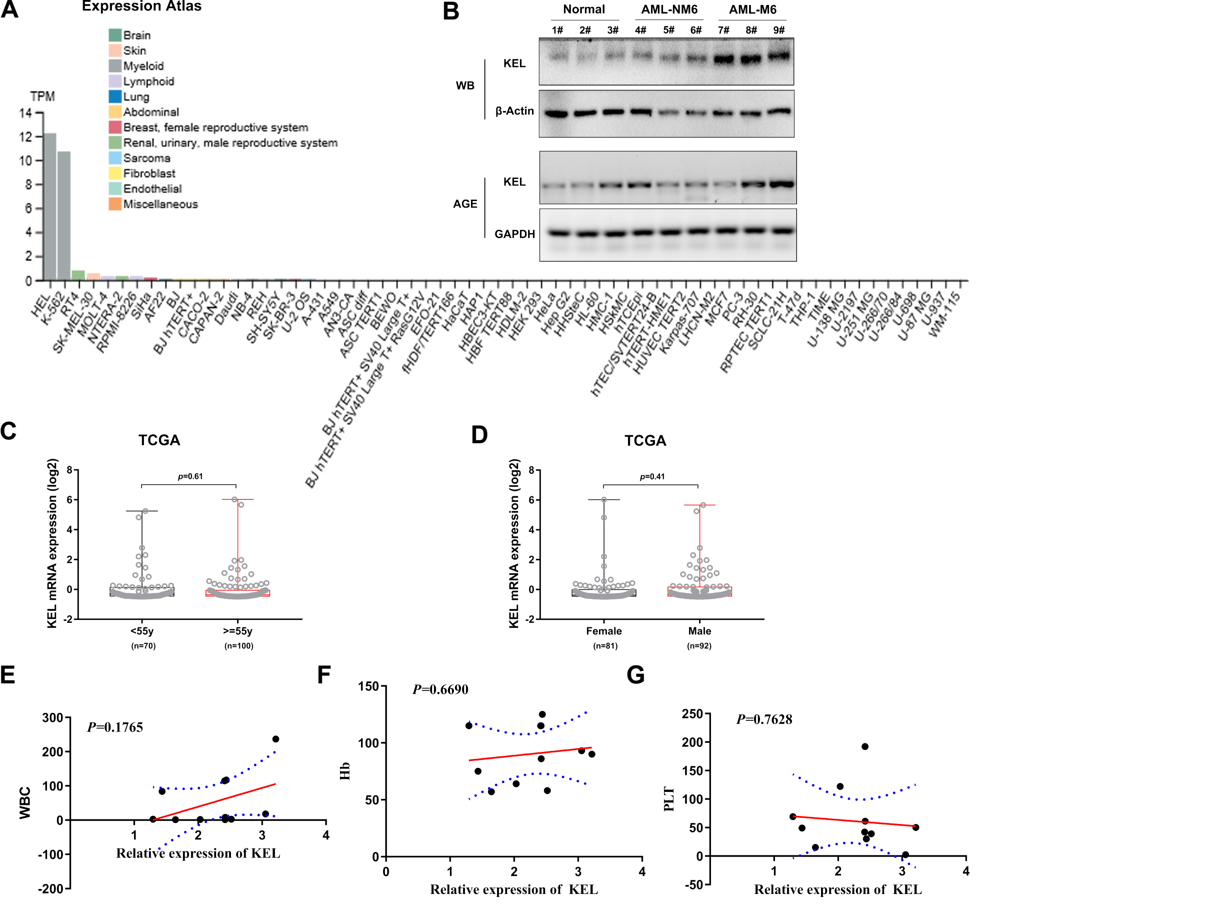

A
Expression Atlas
Brain
Skin
Myeloid
Lymphoid
Lung
Abdominal
Breast, female reproductive system
Renal, urinary, male reproductive system
Sarcoma
Fibroblast
Endothelial
Miscellaneous
TPM
HEL
K-562
RT4
SK-MEL-30
MOLT-4
NTERA-2
RPMI-8226
SiHa
AF22
BJ
BJ hTERT+
CACO-2
CAPAN-2
Daudi
NB-4
REH
SH-SY5Y
SK-BR-3
U-2 OS
A-431
A549
AN3-CA
ASC diff
ASC TERT1
BEWO
BJ hTERT+ SV40 Large T+
BJ hTERT+ SV40 Large T+ RasG12V
EFO-21
fHDF/TERT166
HaCaT
HAP1
HBEC3-KT
HBF TERT88
HDLM-2
HEK 293
HeLa
Hep G2
HHSteC
HL-60
HMC-1
HSkMC
hTCEpi
hTEC/SVTERT24-B
hTERT-HME1
HUVEC TERT2
Karpas-707
LHCN-M2
MCF7
PC-3
RH-30
RPTEC TERT1
SCLC-21H
T-47d
THP-1
TIME
U-138 MG
U-2197
U-251 MG
U-266/70
U-266/84
U-698
U-87 MG
U-937
WM-115
B
Normal
AML-NM6
AML-M6
1# 2# 3# 4# 5# 6# 7# 8# 9#
WB
KEL
β-Actin
AGE
KEL
GAPDH
C
TCGA
KEL mRNA expression (log2)
p=0.61
<55y
(n=70)
>=55y
(n=100)
D
TCGA
KEL mRNA expression (log2)
p=0.41
Female
(n=81)
Male
(n=92)
E
WBC
P=0.1765
Relative expression of KEL
F
Hb
P=0.6690
Relative expression of KEL
G
PLT
P=0.7628
Relative expression of KEL
