# Supplementary material for: Functional Evaluation of KEL as an Oncogenic Gene in the Progression of Acute Erythroleukemia
Source: Oxid Med Cell Longev. 2022 Jan 30;2022:5885342. doi: 10.1155/2022/5885342 (PMC8819426; doi:10.1155/2022/5885342)
Supplement: Supplementary 3 — Additional file: Figure S3: biological and regulatory function of KEL. (A) Benzidine staining of K562 induced with NaBu. (B) Regulatory potential of the GATA1 histone moderation predicted by online database Toolkit for Cistrome Data Browser (http://dbtoolkit.cistrome.org/). (C) ChIP-seq analysis of GATA1 from Toolkit. Design diagram of primers to perform ChIP-qPCR was showed. Data represents the mean ± SD (n = 3). [file 5885342.f3.pdf]

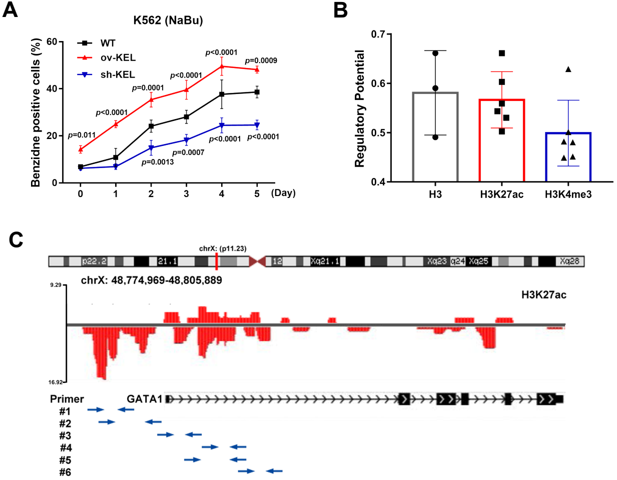
A
K562 (NaBu)
WT
ov-KEL
sh-KEL
Benzidne positive cells (%)
60
40
20
0
0
1
2
3
4
5
(Day)
p=0.011
p<0.0001
p=0.0001
p<0.0001
p<0.0001
p=0.0009
p=0.0013
p=0.0007
p<0.0001
p<0.0001
B
Regulatory Potential
0.7
0.6
0.5
0.4
H3
H3K27ac
H3K4me3
C
chrX: (p11.23)
p22.2
21.1
12
Xq21.1
Xq23
q24
Xq25
Xq28
chrX: 48,774,969-48,805,889
9.29
16.92
H3K27ac
Primer
GATA1
#1
#2
#3
#4
#5
#6
